# A New Mitochondrial Genome of *Sogatella furcifera* (Horváth) (Hemiptera: Delphacidae) and Mitogenome-Wide Investigation on Polymorphisms

**DOI:** 10.3390/insects12121066

**Published:** 2021-11-27

**Authors:** Jongsun Park, Hong Xi, Jonghyun Park, Bo Yoon Seo

**Affiliations:** 1 InfoBoss Research Center, InfoBoss Inc., 301 Room, 670, Seolleung-ro, Gangnam-gu, Seoul 06088, Korea; twinstar@infoboss.co.kr (H.X.); poongeee@infoboss.co.kr (J.P.); 2 Crop Protection Division, National Institute of Agricultural Sciences, RDA, Wanju 55365, Korea

**Keywords:** *Sogatella furcifera*, mitochondrial genome, heteroplasmy, intraspecific variations, simple sequence repeats, phylogenetic analysis

## Abstract

**Simple Summary:**

We completed one mitogenome of white-backed planthopper (WBPH), *Sogatella furcifera* (Horváth), with finding heteroplasmy phenomenon confirmed by PCR reaction and Sanger sequencing method. This heteroplasmy was not observed in WBPHs (*n* = 24) collected from the fields, suggesting that it may be uncommon in fields. We also analyzed single nucleotide polymorphisms, insertion and deletions, and simple sequence repeats among three currently available WBPH mitogenomes of Korea and China, suggesting that identified intraspecific variations could be potential candidates for developing markers to distinguish geographical populations of WBPH including Korean and Chinese. Phylogenetic analysis of 32 mitogenomes of Delphacidae including the three WBPH mitogenomes suggested that Delphacinae seems to be monophyletic and *Sogatella* species including WBPH are clearly formed as one clade.

**Abstract:**

White-backed planthopper (WBPH), *Sogatella furcifera* (Horváth), is one of the major sap-sucking rice pests in East Asia. We have determined a new complete mitochondrial genome of WBPH collected in the Korean peninsula using NGS technology. Its length and GC percentages are 16,613 bp and 23.8%, respectively. We observed one polymorphic site, a non-synonymous change, in the *COX3* gene with confirmation heteroplasmy phenomenon within individuals of WBPH by PCR amplification and Sanger sequencing, the first report in this species. In addition, this heteroplasmy was not observed in wild WBPH populations, suggesting that it may be uncommon in fields. We analyzed single nucleotide polymorphisms, insertion, and deletions, and simple sequence repeats among the three WBPH mitogenomes from Korea and China and found diverse intraspecific variations, which could be potential candidates for developing markers to distinguish geographical populations. Phylogenetic analysis of 32 mitogenomes of Delphacidae including the three WBPH mitogenomes suggested that Delphacinae seems to be monophyletic and *Sogatella* species including WBPH are clearly formed as one clade. In the future, it is expected that complete mitogenomes of individuals of geographically dispersed WBPH populations will be used for further population genetic studies to understand the migration pathway of WBPH.

## 1. Introduction

*Sogatella furcifera* (Horváth), commonly called the white-backed planthopper (WBPH), is an important rice pest species in East Asian rice fields [1,2]. It has usually migrated from the subtropic to temperate regions. For example, they migrate from China to Korea almost every year [1], resulting in this species being listed on the National Species List of Korea [3]. It damages rice plants by feeding directly, causing a decreased filling percentage of kernels and kernel weights [4], and hopper burn is a characteristic symptom of WBPH [5]. Due to the economic importance of this species in rice-cultivating areas [6], whole genome [7], and complete mitochondrial genomes [8] were successfully sequenced and analyzed. These genomic resources of WBPH, which are already available for in-depth analyses, provide a favorable environment for a detailed investigation of its mitochondrial genomes. Because of the regular migration of WBPH from China to Korea, identification of the origin of WBPH has become an important issue for controlling this pest [9,10,11]. One easy way to identify this is by utilizing molecular markers, including mitochondrial genes [12,13,14,15,16]. For example, the investigation of intraspecific variations in the mitochondrial genome of *Laodelphax striatellus* (Fallén) along with the samples distributed in China [17] demonstrated that the two major populations of *L. striatellus*, haplotypes A and B [17].

Next-generation sequencing (NGS) technologies [18,19,20] together with genome assembly programs based on NGS short reads [21,22,23,24,25] have increased the number of sequenced insect mitochondrial genomes. The number of available insect mitochondrial genomes was 6427 as of May 2021, according to the GenomeArchive^®^ [26]. It is more than 48 times the number of insect mitochondrial genomes in 2009 (132 insect complete or near-complete mitochondrial genomes) [27]. In addition, the number of representative mitochondrial genome sequences that NCBI has curated is 2441, indicating that 2441 distinct insect species cover one or more mitochondrial genomes. 

Because of the short length of NGS raw reads, especially those generated by the Illumina HiSeq platform (36 bp–151 bp) [28], the minimum depth for assembly is relatively high, for example, average 30× coverage for human genome resequencing [29], in comparison to that of the Sanger sequencing method: 6×–10× depth for de novo assembly [30,31]. Interestingly, this high coverage of NGS raw reads provides a deep profile of each base of the assembled mitochondrial genomes [32,33,34,35,36,37,38], usually because the amount of mitochondrial genomic DNA in a cell is much higher than that of the main chromosomes. This deep profile can provide the polymorphic bases that display more than one nucleotide in a specific position of the mitochondrial genome. Once these bases demonstrate that alternative nucleotides show a higher proportion than the base calling error rate, they can be potential polymorphic sites or misassemble areas. 

The mitochondrial genome can be highly polymorphic among individuals even in the same species but is usually homogeneous within one individual in insects because it is inherited from the mother’s side [39]. However, different types of mitochondrial genomes in one individual, called mitochondrial heteroplasmy [40], can be found in unusual cases. Interestingly, this mitochondrial heteroplasmy has been reported in insect mitochondrial genomes, including *Drosophila mauritiana* Tsacas & David [41,42], bark weevils [43], *Cimex lectularius* Linnaeus [44], and *Aedes aegypti* (Linnaeus in Hasselquist) [45]. This indicates that many insect species may present with mitochondrial heteroplasmy. However, multiple individuals of small insects including planthoppers, are usually required to extract sufficient genomic DNA for NGS sequencing. Consequently, even though the sample does not exhibit heteroplasmy, the extracted DNA appears to demonstrate heteroplasmy because the DNA sample was derived from multiple individuals, with different sequences of the mitochondrial genome. This indicates that the NGS of small insects has the potential to exhibit a ‘virtual’ heteroplasmy problem. In this case, cross-confirmation of the actual nucleotide sequence by the Sanger sequencing method is required to investigate the mitogenome-wide polymorphism.

Here, we report a new WBPH mitochondrial genome and suggest the phenomenon of mitochondrial heteroplasmy from the assembled mitogenome of a WBPH sample consisting of multiple individuals. We confirmed this heteroplasmy phenomenon in the WBPH mitochondrial genome by comparing sequences of PCR products obtained from each individual sample using the Sanger sequencing method. In addition, we conducted a comparative analysis of WBPH mitogenomes to understand their intraspecific variations as well as simple sequence repeats (SSRs) to prepare the fundamental data to develop molecular markers to distinguish geographical populations of WBPH and to track its region-of-origin of migration. Finally, we carried out a phylogenetic analysis based on the complete mitogenomes of the family Delphacidae available and compared with the previous phylogenetic studies based on several marker sequences [46] to evaluate a newly assembled WBPH mitogenome data in this study. It provides the clear taxonomic relationship between WBPH captured in Korea and 30 species of Delphacidae and one species of Cixiidae.

## 2. Materials and Methods

### 2.1. DNA Extraction of the WBPH Samples

We extracted genomic DNA from a total of 20 WBPH individuals of the Taean population of WBPHs (named as WBPHTA) using the CTAB-based DNA extraction method (iNtRON biotechnology, Inc., Seoungnam-si, Korea) for next-generation sequencing. Each genomic DNA of ten WBPH individuals in WBPHTA and 24 WBPH individuals in two wild WBPH populations was additionally extracted and used to determine nucleotide sequences in a polymorphic site of the *COX3* gene through Sanger sequencing. WBPHTA was captured at paddy fields (N 36°38′, E 126°18′) in Taean-gun in the Republic of Korea in 2006 and has been kept with rice seedlings in the insectary [25 ± 2 °C, 60 ± 5% RH, 14 (L): 10 (D)] of National Institute of Agricultural Sciences in Korea. The two wild populations, named as WBPHHD and WBPHBS, were sampled at paddy fields in Hadong-gun (N 34°58′20″, E 127°50′57″) and Boseong-gun (N 34°50′28″, E 127°16′36″), respectively, in the Republic of Korea on 19 July 2021.

### 2.2. Genome Sequencing and De Novo Assembly of WBPH Mitogenome

Genome sequencing was performed using HiSeqX at Macrogen Inc., Seoul, Korea from extracted DNA of the WBPH sample. De novo assembly and confirmation were done by Velvet v1.2.10 [21] after filtering raw reads using Trimmomatic v0.33 [47] with default parameters. The assembled mitochondrial genome sequence was confirmed with BWA v0.7.17 [48] (alignment of raw reads against the assembled sequence) and SAMtools v1.9 (tview mode for a manual check of each base) [49]. All bioinformatic processes were conducted under the environment of the Genome Information System (GeIS; http://geis.infoboss.co.kr/; 24 November 2021) which have been utilized in the various genomic studies [50,51,52,53].

### 2.3. Annotation of WBPH Mitogenome

Geneious Prime^®^ v2020.2.4 (Biomatters Ltd., Auckland, New Zealand) was used to annotate mitochondrial genome based on the previously sequenced mitogenome of WBPH (NC_021417) [8] with ARWEN [54] for annotating tRNAs.

### 2.4. Verification of Polymorphic Sites on the WBPH Mitogenome Using PCR and Sanger Sequencing Methods

To verify the sequence of one polymorphic site (A/T_4667_) in the whole WBPH mitogenome, we designed a PCR primer set, Sofur-mt4218F (5′-ACA CTA ACC TAA TAT TTG CC-3′) and Sofur-mt4945R (5′-GAT GCT CCT GAT CTT AAT AA-3′). PCR amplification of each genomic DNA for ten WBPH individuals of WBPHTA, eight individuals of WBPHHD, and 16 individuals of WBPHBS was conducted with PrimeSTAR GXL DNA polymerase (Takara Korea Biomedical Inc., Seoul, Korea) by following 30 cycles of 10 s at 98 °C, 15 s at 50 °C, and 1 min at 68 °C. Sager sequencing of PCR products was carried out by the DNA sequencing service of Macrogen Inc. Korea using ABI 3730xl System (Thermo Fisher Scientific, MA, USA).

### 2.5. Prediction of Three-Dimensional Structure of COX3 of WBPH Mitogenome 

To predict the three-dimensional structure of COX3, Swiss-Model (https://swissmodel.expasy.org/; 24 November 2021) [55] was used with the amino acid sequence of COX3. ‘Build Mode’ was used for constructing two types of COX3 amino acids caused by the heteroplasmy we identified in this study. The figure of the three-dimensional structure was also drawn by the web interface of Swiss-Model directly.

### 2.6. Identification of Intraspecific Variations of WBPH Mitogenomes 

Pair-wise sequence alignments among complete mitogenome of WBPH including the mitogenome sequenced in this study were conducted by MAFFT v7.450 [56] with the default option. Identifying intraspecific variations, SNPs and INDELs, on the WBPH mitogenomes was conducted using the function, ‘Find variations/SNPs,’ implemented in the Geneious Prime^®^ v2020.2.4 (Biomatters Ltd., Auckland, New Zealand), which has been utilized in the previous insect mitochondrial genome studies [37,57,58,59,60,61,62,63,64]. When the number of INDELs was calculated, continuous INDEL bases were considered one INDEL region.

### 2.7. Identification of Simple Sequence Repeats on the WBPH Mitogenomes 

Simple sequence repeats (SSRs) were identified on the mitogenome sequence using the pipeline of the SSR database (SSRDB; http://ssrdb.infoboss.co.kr/; 24 November 2021) which has been utilized in the previous studies [65,66,67,68,69,70]. Based on the conventional definition of an SSR on the organelle genome, monoSSR (1 bp) to hexaSSR (6 bp), the total length of SSRs on the chloroplast genome exceeds 10 bp. We used the criteria as follows: the monoSSR (unit sequence length of 1 bp) to hexaSSR (6 bp) are used as normal SSRs, and heptaSSR (7 bp) to decaSSR (10 bp) are defined as extended SSRs. Among the normal SSRs, pentaSSRs (5 bp) and hexaSSRs (6 bp) are classified as potential SSRs.

### 2.8. Comparison of SSRs Identified from the WBPH Mitogenomes 

SSRs identified from the three WBPH mitogenomes were compared based on their flanking sequences under the environment of the SSRDB (http://ssrdb.infoboss.co.kr/; 24 November 2021) to identify intraspecific variations of SSRs. The pipeline of the SSR comparison implemented in the SSRDB used in various organelle genome studies [65,68,69,70] was used with the following conditions: a cut-off e-value of 1 × 10^−10^ and a maximum flanking sequence for the comparison of 60 bp.

### 2.9. Construction of Phylogenetic Trees

The 33 mitogenomes including 32 representative mitogenomes (30 species) selected for each species in the Delphacidae family and one outgroup mitogenome of *Haplaxius crudus* (Van Duzee) in the Cixiidae family [46] were aligned without control region by MAFFT v7.450 [56], and alignment quality was checked manually. The maximum likelihood (ML) tree was reconstructed in IQ-TREE v1.6.6 [71]. In the ML analysis, a heuristic search was used with nearest-neighbor interchange (NNI) branch swapping, TVM + F + R4 model, and uniform rates among sites suggested by the model finder implemented in IQ-TREE. All other options used the default settings. Bootstrap analyses with 1000 pseudoreplicates were conducted with the same options. The posterior probability of each node was estimated by Bayesian inference (BI) using the MrBayes v3.2.6 [72] plug-in implemented in Geneious Prime^®^2020.2.4 (Biomatters Ltd., Auckland, New Zealand). The HKY85 model with gamma rates was used as a molecular model. A Markov chain Monte Carlo algorithm was employed for 1,100,000 generations, sampling trees every 200 generations, with four chains running simultaneously. Trees from the first 100,000 generations were discarded as burn-in.

## 3. Results and Discussions

### 3.1. Complete Mitochondrial Genome of S. furcifera

**Figure 1:**
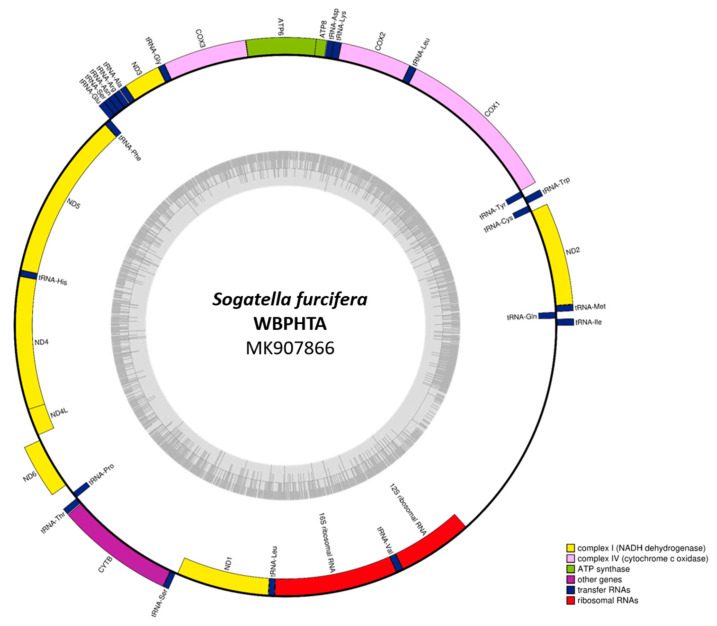
Complete WBPHTA mitochondrial genome sequence. Black circle indicates the WBPHTA mitogenome, yellow bars are protein-coding genes, purple bars are tRNAs, and red bars mean rRNAs. Each gene name was displayed with lines directing to the corresponded bars.

In this study, we present the complete mitochondrial genome of *S. furcifera* (WBPHTA; GenBank accession number: MK907866) with a length of 16,613 bp, containing 13 protein-coding genes (PCGs), 22 transfer RNAs (tRNAs), and 2 ribosomal RNAs (rRNAs) (Supplementary Table S1 and Figure 1), which is a typical configuration of insect mitochondrial genomes [73,74]. It is similar to one of the mitogenomes previously sequenced (WBPHHN; GenBank accession number: NC_021417; 16,612 bp; Table 1). The GC percentage of the WBPHTA mitogenome was 23.8%, which is the same as the previously sequenced WBPH mitogenomes (WBPHHN and WBPHYN; Table 1).

The AT-rich region, considered as a putative control region of the WBPHTA mitogenome, is 2219 bp long, which is the shortest length among the three WBPH mitogenomes (Table 1). The variations found in a putative control region mostly contributed to the length difference of WBPH mitogenomes (see the section, Intraspecific mitochondrial variations compared with the previously sequenced WBPH mitogenomes). Usually, variations of this putative control region are larger than those in the remaining regions [65], therefore, it can be utilized as a good genetic marker [75]. The GC-skew and AT-skew of the WBPHTA mitogenome are calculated as −0.14163 and 0.094715, respectively, similar to those of the other two WBPH mitogenomes (Table 1).

**Figure 2:**
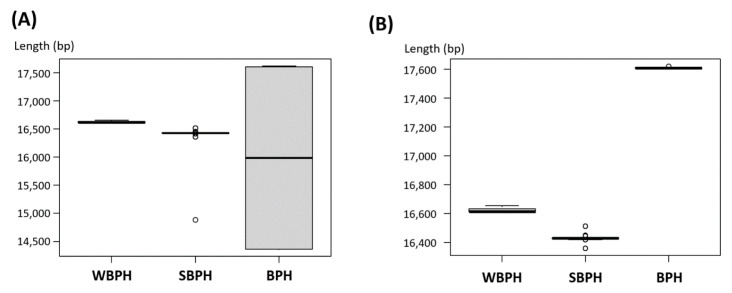
Length variations of the WBPH, SBPH, and BPH complete mitogenomes. (**A**) present the histogram of all available WBPH, SBPH, and BPH complete mitogenomes. The grey-colored box indicates 75 percentile of mitogenome length and the middle line means the median value of mitogenome length. Small circles indicate outliers. (**B**) display the histogram of all available WBPH, SBPH, and BPH mitogenomes except the seven BPH mitogenomes which lack their control regions.

The length variations of complete mitogenomes of two planthopper species, the brown planthopper (BPH; *Nilaparvata lugens* (Stål)) [57,60,76,77,78] and the small brown planthopper (SBPH; *L. striatellus*) [63,77,79], were investigated (Figure 2A). Interestingly, the length variations of SBPH and BPH complete mitogenomes were approximately 1.5 kb and 3 kb, respectively, different from that of WBPH (i.e., less than 100 bp; Figure 2A). One SBPH (GenBank accession number: MW732715; unpublished) and five BPH complete mitogenomes [57,60,76,77,78] were significantly shorter than those of the remaining mitogenomes, due to the short length of the control region, or lack of this region. We speculate that these six mitogenomes might lack the control region, even though they were labeled as complete mitogenomes, because of difficulties of assembly. After removing these six short complete mitogenomes, SBPH exhibits the largest variations because of the large number (86) of complete mitogenomes [63,77,79], while the remaining two species exhibited a small range of length variations (Figure 2B), indicating that length variations of planthoppers can be affected by the number of mitogenome sequences obtained from individuals of various geographical regions.

### 3.2. Identification and Verification of the Polymorphic Site Found from the WBPH Mitogenome

**Figure 3:**
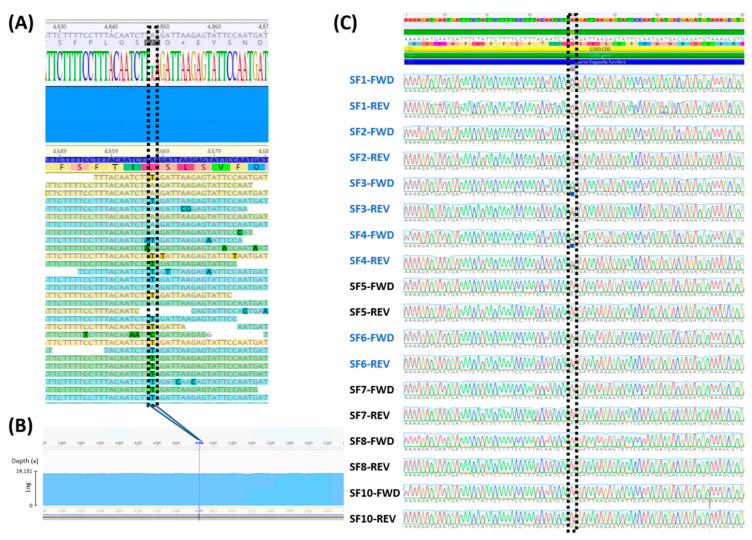
Identification and validation of polymorphic site on WBPHTA mitogenome. (**A**) presents the alignment of NGS raw reads around the polymorphic site on the WBPHTA mitogenome (4667 bp). Consensus bases were displayed as colored base characters. Assembled mitogenome sequences were presented with coordination and coded amino acids. NGS raw reads aligned against the assembled mitogenome were displayed under the assembled mitogenome sequences. The block-dotted box indicates the polymorphic site. (**B**) exhibited sequencing depth around the heteroplasmy site (4667th). The *X*-axis indicates coordination of mitogenome and *Y*-axis presented sequencing depth as log scale. (**C**) shows the alignment of sequencing chromatograms against the WBPHTA mitogenome. Labels at the left side indicate sample name (SF plus number) and sequencing direction (FWD; forward and REV; reverse) together. The block-dotted box indicates the polymorphic site.

During the base confirmation process of the assembled WBPH mitogenome, we found that one base of *COX3* at 4667th starting from *tRNA-Ile* on the mitogenome, presented A and T together (Figure 3A). The ratio of A to T was close to 1:1 (Figure 3A), indicating that this site may have two different bases. In addition, sequencing depth around the region of 4667th nucleotide displayed around 19,000× depth without any duplication signal in this region (Figure 3B). This phenomenon can be explained by the fact that 20 WBPH individuals were used for extracting DNA (see Materials and Methods).

To confirm whether this polymorphic site contains both bases, we amplified the region around this site of the ten additional individuals (SF1 to SF10) by PCR and sequenced the PCR products using the Sanger sequencing method. We obtained the sequencing results from nine out of ten individuals (Figure 3B), resulting in five out of nine individuals, SF1, SF2, SF3, SF4, and SF6, presenting the polymorphic base, A and T, simultaneously within one individual (Figure 3B). Because of this polymorphic base, base calling results at this site from the five individuals were not consistent (Figure 3B). This result is different from what we expected, namely, that heterogeneous haplotypes from multiple individuals can cause a ‘virtual’ heteroplasmy phenomenon based on NGS results. However, there was a real heteroplasmy phenomenon in WBPH individuals, and this is the first report of heteroplasmy in the mitochondrial genome of a species in the family Delphacidae.

Because this heteroplasmy phenomenon was only observed in WBPH individuals from WBPHTA, a breeding line, has been established and reared in the insectary since 2006, it was necessary to evaluate whether this heteroplasmy in the WBPH mitogenome is also common in wild populations or not. A 4667th nucleotide sequence of *COX3* from a total of 24 individuals of the two Korean wild WBPH populations, WBPHHD and WBPHBS, was investigated using PCR and the Sanger sequencing method. In the results, there was no heteroplasmy phenomenon and single nucleotide polymorphism (SNP; only A) at the 4667th of the WBPH mitogenome. It suggests that the heteroplasmy and SNP of WBPHTA mitogenome might be uncommon in the wild WBPH populations in fields. The heteroplasmy of WBPHTA mitogenome would be associated with continuous pressures from the inbreeding environment in the laboratory. It is remained to study whether this heteroplasmy phenomenon of WBPH is artificially created through continuous insect rearing indoors.

**Figure 4:**
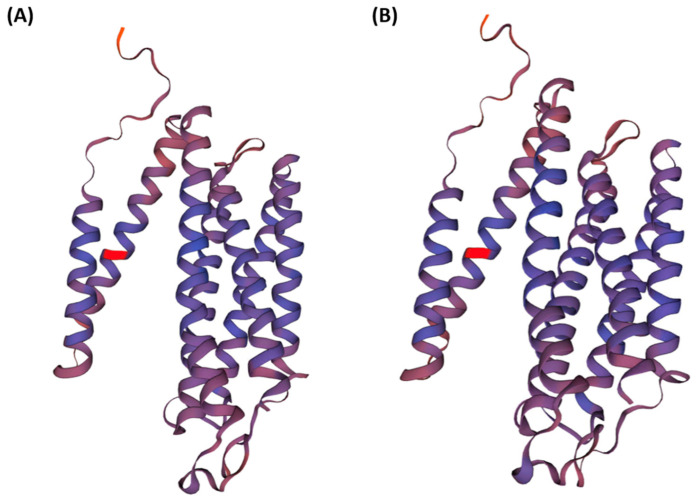
Three-dimensional structure of COX3 with a non-synonymous mutation from heteroplasmy. A three-dimensional structure of *COX3* as ribbon type was displayed. The red color indicates amino acid variation identified from the heteroplasmy phenomenon of WBPH mitogenomes. (**A**) The normal type of *COX3;* (**B**) Mutation type of *COX3* is caused by the heteroplasmy phenomenon identified in this study.

An amino acid substitution from lysine (L) to phenylalanine (F) in *COX3* was deduced by changing A to T at the 4667th position. Moreover, there was one HexaSSR, H0000007 (Supplementary Table S2), which covered this polymorphic site. Once it changes from A to T, the SSR disappears. To evaluate the effectiveness of the three-dimensional structure of *COX3* of non-synonymous SNP, we build the two models using the SWISS-MODEL [55] based on the translated amino acids of the two *COX3* genes. The three-dimensional structure of *COX3* of WBPH exhibited the structure of the seven alpha helixes (Figure 4), which is the same as the three-dimensional structure of the template sequence (2occ.1) with different spatial configurations. The amino acid at the 49th position of *COX3* in both forms did not affect their alpha-helix structure (See red parts in Figure 4A,B), indicating that this non-synonymous mutation from heteroplasmy may not affect three-dimensional structure severely.

The heteroplasmy in the mitogenome has been found in various insect and mite species, including the bed bug (*Cimex lectularius* Linnaeus, Hemiptera:Cimicidae) [80], honeybee (*Apis mellifera* Linnaeus, Hymenoptera:Apidae) [81], a neotropical ant species (*Ectatomma ruidum* (Roger), Formicidae:Ectatomminae) [82], *Anapodisma miramae* Dovnar-Zapolskij (Orthoptera:Acrididae) [83], *Tetrodontophora bielanensis* (Waga) (Poduromorpha:Onychiuridae) [84], and *Drosophila melanogaster* Meigen (Diptera:Drosophilidae) and is caused by paternal mitochondrial DNA leakage [85,86]. It has been found that the heteroplasmy phenomenon in the mitogenome can be involved in biological functions, including pesticide resistance [87,88,89,90] and xenobiotics detoxification [91]. Taken together, our finding suggests that further investigation is needed to determine whether this mitochondrial heteroplasmy in WBPH has biological meanings, such as its physiological mechanism. Moreover, once insect mitogenomes assembled by NGS raw reads displayed any heterogeneous bases, they have to be validated using the other experimental methods whether these bases can be candidates of real heteroplasmy or not.

### 3.3. Intraspecific Mitochondrial Variations Compared with the Previously Sequenced WBPH Mitogenomes

**Figure 5:**
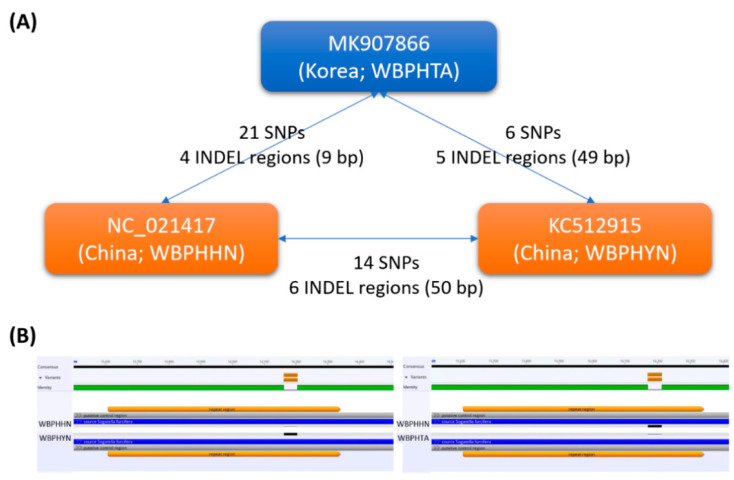
Intraspecific variations were identified from the three WBPH mitogenomes. (**A**) The blue-colored round box indicates WBPHTA mitogenome and two yellow-colored round boxes mean the two previously sequenced WBPH mitogenomes. Numbers of SNPs and INDELs with the total length of INDELs identified from the two WBPH mitogenomes are presented on the lines connected to the boxes. (**B**) Diagram of the longest INDEL identified among three WBPH mitogenomes.

Based on three pair-wise alignments of WBPH mitogenomes, 21 SNPs and four insertions and deletion (INDEL) regions (9 bp in total) and six SNPs and five INDEL regions (49 bp in total) against WBPHHN and WBPHYN mitogenomes were identified (Figure 5A). The longest INDEL found between the WBPHYN and WBPHTA mitogenomes were 42 bp in length in the control region. The control region is the most variable region in insect mitogenomes [65,75,92], which can explain the INDEL identified in the control region. In addition, 14 SNPs and six INDEL regions (50 bp in total) were also found between the WBPHHN and WBPHYN mitogenomes, displaying the same 42-bp INDEL between the two mitogenomes (Figure 5B).

Thirteen of 21 SNPs (61.90%) between the WBPHTA and WBPHHN mitogenomes, six of six SNPs (100.00%) between the WBPHTA and WBPHYN mitogenomes, and 11 of 14 SNPs (78.57%) between the WBPHHN and WBPHYN mitogenomes were found in PCGs. Eight of 13 PCGs, including *ND2*, *COX1*, *COX3*, *ND3*, *ND5*, *ND4*, *CytB*, and *ND1*, contained one or more SNPs. Four of eight PCGs, *COX3*, *ND5*, *ND4*, and *CytB*, contained one non-synonymous SNP in each pair of WBPH mitogenomes. These non-synonymous SNPs can affect the function of the PCGs as reported for *L. striatellus* [93]. These PCGs can be a potential target to develop molecular markers to distinguish populations of WBPHs once more mitogenomes of WBPHs isolated from different locations are available.

The numbers of SNPs and INDEL regions identified from the three WBPH mitogenomes were relatively smaller than those of *L. striatellus* [63,79], *N. lugens* [76], and *Chilo supressalis* (Walker) [62], of which samples were captured in Korea and China, indicating that the genetic diversity of WBPH might be lower than those of the three species. Furthermore, the numbers of intraspecific variations in WBPHs were also smaller than those of *Aphis gossypii* Glover [61,94,95] and *Spodoptera frugiperda* (J. E. Smith) [64] but were larger in number than those of Korean samples of *Hipparchia autonoe* (Esper) [96] and *Alphitobius diaperinus* Panzer [58].

### 3.4. Identification and Comparison of Simple Sequence Repeats on WBPH Mitogenomes

**Figure 6:**
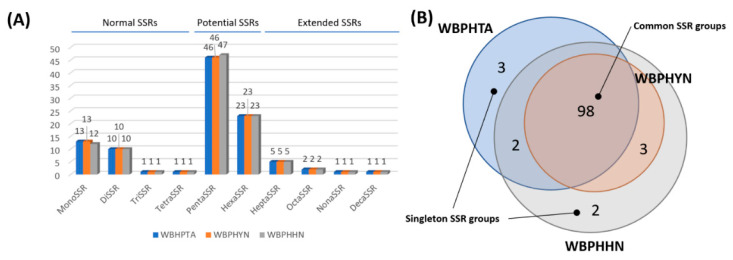
SSR comparison was identified from the three WBPH mitogenomes. (**A**) The *X*-axis indicates SSR types and *Y*-axis shows the number of SSRs. Three colors, blue, orange, and grey, mean the three WBPH mitogenomes, WBPHHN, WBPHYN, and WBPHTA, respectively. (**B**) Vann diagram displays the classification of SSR groups identified from the three WBPH mitogenomes. Transparent blue-colored, red-colored, and grey-colored circles indicate the mitogenome of WBPHTA, WBPHHN, and WBPHYN, respectively. Numbers on the Vann diagram present a number of SSR groups.

Simple sequence repeats (SSRs), which can be utilized as molecular markers to distinguish insect species [97,98] and to identify cryptic insect species [99], were identified from the three WBPH mitogenomes. The WBPHTA and WBPHHN mitogenomes displayed 25 normal SSRs, 69 potential SSRs, and 9 extended SSRs, whereas the WBPHHN mitogenome exhibited 24 normal SSRs (Table 2), 70 potential SSRs, and 9 extended SSRs (Figure 6A; Supplementary Table S2). The differences in the number of monoSSRs and pentaSSRs among the three mitogenomes were one (Figure 6A). This intraspecific difference in SSRs is similar to that of *Stegobium paniceum* (Linnaeus, 1758) (one difference in monoSSR, diSSR, and hexaSSR; Park et al., under review) and *Monomorium pharaonis* (Linnaeus, 1758) (one difference in pentaSSR and decaSSR; Park et al., under revision).

Comparisons of flanking sequences of each SSR identified from the three WBPH mitogenomes using the SSRDB (see Materials and Methods), identified 108 SSR groups: 98 out of 108 SSR groups (90.74%) contained three SSRs originating from three WBPH mitogenomes, called common SSRs (Figure 6B). Three of the five SSR groups (60.00%), covering two SSRs from the two mitogenomes, contained SSRs of WBPHHN and WBPHYN, captured in China, while two of the five SSR groups (40.00%) were from WBPHHN and WBPHTA (Table 3; Figure 6B). Two of the five SSR groups consisting of the two Chinese WBPHs were located in the *CTYB* gene (SSRGroup 75 and SSRGroup 103; Table 3), whereas SSRGroup 34 contains the monoSSRs originating from WBPHHN and WBPHTA (Table 3). Furthermore, singleton SSR groups consisted of two SSRs from WBPHHN and three SSRs from WBPHTA (Table 3; Figure 6B). All five singleton SSRs were in the intergenic regions: (i) the region between *ND6* and *tRNA-Pro* and (ii) the putative control region (Table 3). Interestingly, WBPHYN did not have any singleton SSR (Figure 6B), indicating that the WBPHYN mitogenome does not have any unique SNPs or INDELs in SSRs. The ratio of singleton SSRs of WBPH mitogenomes was similar to that of *M. pharaonis* (7.00%; Park et al., under revision) but was lower than that of *S. paniceum* (12.85%; Park et al., under review).

The SSRs identified from the three WBPH mitogenomes were densely distributed in the putative control regions as well as in the *ND4* and *ND1* genic regions. Interestingly, the 5′ part of the putative control region displayed a high density of SSRs in a conserved manner across the three mitogenomes, while the 3′ part of the putative control regions exhibited a low density of SSRs with variable manners across the three mitogenomes). This correlation between SSR density and intraspecific variations will be investigated more in other insect species, which can be used as a guide for developing efficient molecular markers using SSRs. The intraspecific SSRs identified in this study could be potential candidates for developing markers with WBPH mitogenomes which will be sequenced in near future to distinguish geographical populations and to understand the migration pathway of WBPH.

### 3.5. Phylogenetic Tree of 32 Delphacidae Mitogenomes including Three WBPH Mitogenomes

**Figure 7:**
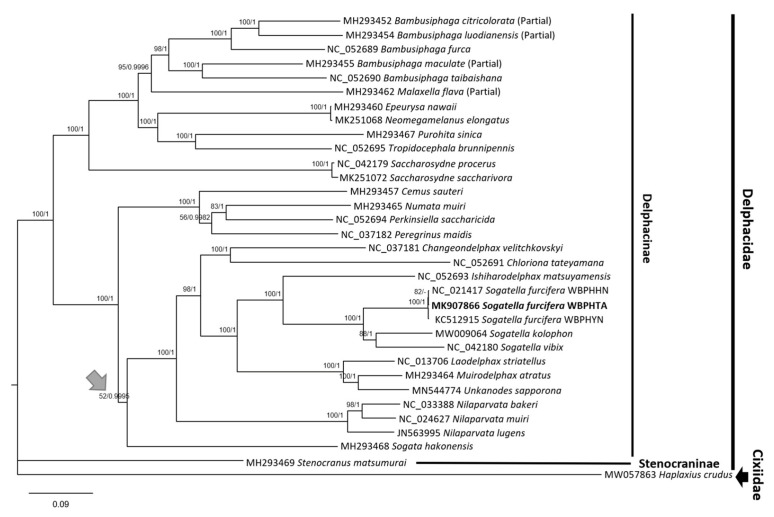
Phylogenetic tree of 32 Delphacidae mitogenomes. Phylogenetic trees constructed by Maximum-Likelihood (ML) and Bayesian inference (BI) methods were presented with supportive values of ML and BI, respectively, on the branches. Accession names and species names were printed on the right side of the phylogenetic tree. Subfamily names were shown with the thick bars at the right side of the tree.

Maximum-likelihood (ML) and Bayesian inference (BI) phylogenetic trees of 32 mitogenomes of Delphacidae including three WBPH mitogenomes and one outgroup species, *Haplaxius crudus* van Duzee, 1907, were constructed. Among the 32 Delphacidae mitogenomes, only one mitogenome, *Stenocranus matsumurai* Metcalf, 1943(GenBank accession number: MH293469), belongs to the subfamily Stenocraninae, while the remaining 31 mitogenomes belong to the subfamily Delphacinae. Phylogenetic trees demonstrated that both subfamilies were clearly distinct from each other (Figure 7). In addition, all branches except one (see grey arrow in Figure 6) were supported by the high supportive values of both trees (Figure 1), displaying a rigid phylogenetic relationship within the family Delphacidae. This is congruent with the previous phylogenetic studies [46,100,101], supporting that the phylogenetic relationship of the 30 Delphacidae species become more supportive in comparison to the incongruent cases of molecular phylogeny based on between marker sequences and complete mitogenomes [102,103,104]. The exceptional clade displaying low supportive values of ML (52; Figure 7) was also presented lower supportive values of both ML and BI trees (Clade III) in the previous phylogenetic study [46]. However, the bootstrap value of BI was increased to 0.9995 (Figure 7), indicating that complete mitogenome contributed increment of supportive values in the phylogenetic tree. If more complete mitogenomes of neighbor genera of *Sogata*, such as *Miranus*, *Hadeodelphax*, *Liburniella*, and *Neometopina*, are available in the future, the phylogenetic relationship of this clade is expected to become clearer.

The BI tree demonstrated that three WBPH mitogenomes were clustered in one clade; while the ML tree demonstrated that WBPHTA and WBPHHN were clustered together, suggesting the possibility that the WBPHTA sample may be from a similar region to the Hainan province in China, where the WBPHHN isolate was captured [8], together with the fact that WBPH cannot overwinter in the Korean peninsula [1]. Moreover, the population structure of WBPHs isolated in Yunnan and Shandong provinces in China and five Southeast Asian countries including the Greater Mekong subregion (GMS) are considered to be important overwintering sites, displayed one group regardless of geographical distributions by principal coordinates analysis with a 635-bpmitochondrial *COI* sequence data [14]. Further studies with more mitogenomes of WBPHs are required to determine the origin of Korean WBPHs as well as the immigration routes of WBPH in the Northeast Asian area. Complete mitogenomes of geographically dispersed WBPH populations will be one of the potential data for further population genetic studies to understand the WBPH migration pathway in Asian countries.

## 4. Conclusions

In this study, we reported a new complete mitogenome of WBPH collected in Korea (named as WBPHTA) and firstly identified heteroplasmy in WBPH and Delphacidae. The heteroplasmy resulted in the amino acid substitution in *COX3*, causing not severe conformational change based on the prediction of three-dimensional structure. In addition, this heteroplasmy was not found in the two wild populations in Korea, suggesting that continuous selection pressure may cause this heteroplasmy. Intraspecific variations and simple sequence repeats (SSRs) were identified among the three WBPH mitogenomes, suggesting that these can be a potential target to develop molecular markers to distinguish populations of WBPHs from different locations. Phylogenetic analysis suggested that Delphacinae is monophyletic and WBPH, as one group, is clearly divided with closely related *Sogatella* species. The heteroplasmy, the firstly reported in WBPH mitogenome, suggests an additional step to check the polymorphic sites during the assembly. In addition, intraspecific variations in WBPH mitogenomes can be useful to understand its genetic diversity as well as to develop useful markers and to understand the migration of geographically dispersed WBPH populations.

## Figures and Tables

**Table 1:** Characteristics of three available WBPH mitogenomes.

Name	Length (bp)	GC Ratio (%)	GC Skew	AT Skew	AT-Rich Region Length (bp)	GC Ratio of AT-Rich Region (%)	GenBank Accession Number	Reference
WBPHTA	16,613	23.8	−0.14163	0.094715	2219	17.5	MK907866	This study
WBPHHN	16,612	23.8	−0.14055	0.092920	2223	17.5	NC_021417	[8]
WBPHYN	16,654	23.8	−0.14070	0.094262	2264	17.7	KC512915	[8]

**Table 2:** List of simple sequence repeats (SSRs) identified from a new WBPH mitogenome collected in Korea.

No	Name	SSRType	Type	Start	End	Unit Sequence	Repeat #	Genes
1	M0000001	Normal SSR	MonoSSR	858	868	A	11	ND2
2	M0000002	Normal SSR	MonoSSR	7044	7053	A	10	ND5
3	M0000003	Normal SSR	MonoSSR	7725	7737	A	13	ND5
4	M0000004	Normal SSR	MonoSSR	7830	7839	A	10	ND4
5	M0000005	Normal SSR	MonoSSR	8110	8123	A	14	ND4
6	M0000006	Normal SSR	MonoSSR	8412	8421	A	10	ND4
7	M0000007	Normal SSR	MonoSSR	8717	8727	A	11	ND4
8	M0000008	Normal SSR	MonoSSR	9057	9066	A	10	ND4
9	M0000009	Normal SSR	MonoSSR	10,023	10,032	T	10	
10	M0000010	Normal SSR	MonoSSR	10,033	10,064	A	32	
11	M0000011	Normal SSR	MonoSSR	11,819	11,830	A	12	ND1
12	M0000012	Normal SSR	MonoSSR	14,555	14,569	T	15	
13	M0000013	Normal SSR	MonoSSR	14,966	14,988	T	23	
14	D0000001	Normal SSR	DiSSR	14,785	14,796	TA	6	
15	D0000002	Normal SSR	DiSSR	14,862	14,873	TA	6	
16	D0000003	Normal SSR	DiSSR	15,106	15,115	AT	5	
17	D0000004	Normal SSR	DiSSR	15,147	15,160	AT	7	
18	D0000005	Normal SSR	DiSSR	15,192	15,201	AT	5	
19	D0000006	Normal SSR	DiSSR	15,208	15,223	TA	8	
20	D0000007	Normal SSR	DiSSR	15,244	15,253	TA	5	
21	D0000008	Normal SSR	DiSSR	15,274	15,285	TA	6	
22	D0000009	Normal SSR	DiSSR	15,335	15,344	TA	5	
23	D0000010	Normal SSR	DiSSR	15,387	15,396	TA	5	
24	T0000001	Normal SSR	TriSSR	15,117	15,128	ATT	4	
25	Te0000001	Normal SSR	TetraSSR	8443	8454	ATAA	3	ND4

**Table 3:** List of simple sequence repeats (SSRs) from SSR groups containing one or two mitogenomes.

No	SSR Group	Mitogenome	SSR Type	Type	SSR Name	Start	End	Unit Sequence	# of Repeats	Genes
1	SSRGroup 16	WBPHHN	Potential SSR	PentaSSR	P0000047	15,346	15,355	AATAA	2	Intergenic
		WBPHYN	Potential SSR	PentaSSR	P0000047	15,346	15,355	AATAA	2	Intergenic
2	SSRGroup 34	WBPHHN	Normal SSR	MonoSSR	M0000009	10,023	10,032	T	10	Intergenic
		WBPHTA	Normal SSR	MonoSSR	M0000009	10,023	10,032	T	10	Intergenic
3	SSRGroup 75	WBPHHN	Potential SSR	PentaSSR	P0000023	10,333	10,342	TACAC	2	CYTB
		WBPHYN	Potential SSR	PentaSSR	P0000023	10,333	10,342	TACAC	2	CYTB
4	SSRGroup 99	WBPHYN	Potential SSR	PentaSSR	P0000044	15,034	15,043	ATATA	2	Intergenic
		WBPHTA	Potential SSR	PentaSSR	P0000044	15,035	15,044	ATATA	2	Intergenic
5	SSRGroup 103	WBPHYN	Potential SSR	HexaSSR	H0000021	10,498	10,509	TAAAAG	2	CYTB
		WBPHHN	Potential SSR	HexaSSR	H0000021	10,498	10,509	TAAAAG	2	CYTB
6	Singleton 1	WBPHHN	Potential SSR	PentaSSR	P0000022	10,016	10,025	AATTT	2	Intergenic
7	Singleton 2	WBPHHN	Potential SSR	PetanSSR	P0000044	15,034	15,043	ATATA	2	Intergenic
8	Singleton 3	WBPHTA	Potential SSR	PentaSSR	P0000023	10,337	10,346	TACAC	2	Intergenic
9	Singleton 4	WBPHTA	Potential SSR	PentaSSR	P0000047	15,347	15,356	AATAA	2	Intergenic
10	Singleton 5	WBPHTA	Potential SSR	HexaSSR	H0000021	10,502	10,513	TAAAAG	2	Intergenic

## Data Availability

Mitochondrial genome sequence used in this study can be accessed via accession number MK907866 in NCBI GenBank.

## Supplementary Materials

The following are available online at https://www.mdpi.com/article/10.3390/insects12121066/s1, Table S1: Summary on a new mitochondrial genome of *Sogatella furcifera* collected in Korea, Table S2: List of potential and extended SSRs identified in the WBPHTA mitogenome.
