# Supplementary material for: A New Mitochondrial Genome of Sogatella furcifera (Horváth) (Hemiptera: Delphacidae) and Mitogenome-Wide Investigation on Polymorphisms
Source: Insects. 2021 Nov 27;12(12):1066. doi: 10.3390/insects12121066 (PMC8706918; doi:10.3390/insects12121066)
Supplement: Supplementary file 1 [file insects-12-01066-s001.zip › insects-1378374-supplementary.pdf]

Supplementary Table S1. Summary on a new mitochondrial genome of *Sogatella furcifera* collected in Korea.

Name	Strand	Position (bp)		Size (bp)	Anticodon	Start codon	Stop codon	Intergenic nucleotide (bp)
tRNA-Ile	Y	1	66	66	GAU			N/A
tRNA-Gln	N	69	134	66	UUG			2
tRNA-Met	Y	134	195	62	CAU			-1
ND2	Y	196	1,152	957		ATT	TAA	0
tRNA-Cys	N	1,151	1,215	65	GCA			-2
tRNA-Trp	Y	1,234	1,300	67	UCA			18
tRNA-Tyr	N	1,311	1,373	63	GUA			10
COX1	Y	1,375	2,908	1534		ATG	T	1
tRNA-Leu	Y	2,909	2,973	65	UAA			0
COX2	Y	2,974	3,637	664		ATT	T	0
tRNA-Lys	Y	3,638	3,708	71	CUU			0
tRNA-Asp	Y	3,709	3,768	60	GUC			0
ATP8	Y	3,771	3,872	102		ATT	TAA	2
ATP6	Y	3,866	4,520	655		ATG	T	-7
COX3	Y	4,521	5,301	781		ATG	T	0
tRNA-Gly	Y	5,302	5,363	62	UCC			0
ND3	Y	5,364	5,712	349		ATT	T	0
tRNA-Ala	Y	5,713	5,773	61	UGC			0
tRNA-Arg	Y	5,778	5,838	61	UCG			4
tRNA-Asn	Y	5,838	5,900	63	GUU			-1
tRNA-Ser	Y	5,901	5,954	54	GCU			0
tRNA-Glu	Y	5,955	6,017	63	UUC			0
tRNA-Phe	N	6,018	6,085	68	GAA			0
ND5	N	6,086	7,760	1675		GTG	T	0
tRNA-His	N	7,761	7,820	60	GUG			0
ND4	N	7,821	9,143	1323		ATG	TAA	0
ND4L	N	9,137	9,409	273		ATG	TAA	-7
ND6	Y	9,459	9,968	510		ATT	TAA	49
tRNA-Pro	N	10,066	10,127	62	UGG			97
tRNA-Thr	Y	10,131	10,193	63	UGU			3
CYTB	Y	10,198	11,299	1102		ATG	T	4
tRNA-Ser	Y	11,300	11,355	56	UGA			0

ND1	N	11,373	12,290	918		ATG	TAG	17
tRNA-Leu	N	12,292	12,353	62	UAG			1
l-rRNA	N	12,354	13,578	1225		ACA	T	0
tRNA-Val	N	13,579	13,647	69	UAC			0
s-rRNA	N	13,648	14,394	747		GAA	TAG	0
A-T rich	-	14,395	16,613	2,219	-	-	-	0

Supplementary Table S2. List of potential and extended SSRs identified in the WBPHTA mitogenome.

No	Name	SSRType	Type	Start	End	Unit sequence	Repeat #	Genes
26	P0000001	Potential SSR	PentaSSR	46	55	AAAAT	2	
27	P0000002	Potential SSR	PentaSSR	195	204	AATTA	2	ND2
28	P0000003	Potential SSR	PentaSSR	1423	1432	TATTT	2	COX1
29	P0000004	Potential SSR	PentaSSR	2278	2287	TACTT	2	COX1
30	P0000005	Potential SSR	PentaSSR	3082	3091	ATTTC	2	COX2
31	P0000006	Potential SSR	PentaSSR	3600	3609	AATTA	2	COX2
32	P0000007	Potential SSR	PentaSSR	3712	3721	AGTTT	2	
33	P0000008	Potential SSR	PentaSSR	5362	5371	AAATT	2	ND3
34	P0000009	Potential SSR	PentaSSR	5496	5505	TTCAA	2	ND3
35	P0000010	Potential SSR	PentaSSR	6107	6116	AAAAT	2	ND5
36	P0000011	Potential SSR	PentaSSR	6442	6451	ACATA	2	ND5
37	P0000012	Potential SSR	PentaSSR	6579	6588	AAAAT	2	ND5
38	P0000013	Potential SSR	PentaSSR	7599	7608	CCAAT	2	ND5
39	P0000014	Potential SSR	PentaSSR	7791	7800	AAAAT	2	
40	P0000015	Potential SSR	PentaSSR	8011	8020	AAAAG	2	ND4
41	P0000016	Potential SSR	PentaSSR	8362	8371	AGTAA	2	ND4
42	P0000017	Potential SSR	PentaSSR	8423	8432	AATTT	2	ND4
43	P0000018	Potential SSR	PentaSSR	8794	8803	ACTCT	2	ND4
44	P0000019	Potential SSR	PentaSSR	9143	9152	TAAAT	2	ND4,ND4L
45	P0000020	Potential SSR	PentaSSR	9370	9379	AAATT	2	ND4L
46	P0000021	Potential SSR	PentaSSR	9988	9997	TTAAA	2	
47	P0000023	Potential SSR	PentaSSR	10337	10346	TACAC	2	CYTB
48	P0000024	Potential SSR	PentaSSR	10711	10720	TTCTT	2	CYTB
49	P0000025	Potential SSR	PentaSSR	10724	10733	ATTTT	2	CYTB
50	P0000026	Potential SSR	PentaSSR	10737	10746	TTTTA	2	CYTB
51	P0000027	Potential SSR	PentaSSR	11335	11344	AAATT	2	
52	P0000028	Potential SSR	PentaSSR	11625	11634	AAAAG	2	ND1
53	P0000029	Potential SSR	PentaSSR	11732	11741	AAAAT	2	ND1
54	P0000030	Potential SSR	PentaSSR	11803	11812	ATTAA	2	ND1
55	P0000032	Potential SSR	PentaSSR	12003	12012	AATTA	2	ND1
56	P0000033	Potential SSR	PentaSSR	12796	12805	AAATT	2	
57	P0000034	Potential SSR	PentaSSR	12814	12823	TAATA	2	
58	P0000035	Potential SSR	PentaSSR	13181	13190	AAATT	2	
59	P0000036	Potential SSR	PentaSSR	13307	13316	TTTTC	2	
60	P0000037	Potential SSR	PentaSSR	13390	13399	AAACT	2	

61	P0000038	Potential SSR	PentaSSR	14003	14012	AATTT	2	
62	P0000039	Potential SSR	PentaSSR	14098	14107	AATAT	2	
63	P0000040	Potential SSR	PentaSSR	14243	14252	ATTAA	2	
64	P0000041	Potential SSR	PentaSSR	14574	14583	ACTCA	2	
65	P0000042	Potential SSR	PentaSSR	14600	14609	AATTT	2	
66	P0000043	Potential SSR	PentaSSR	15001	15010	ATTTA	2	
67	P0000044	Potential SSR	PentaSSR	15035	15044	ATATA	2	
68	P0000045	Potential SSR	PentaSSR	15176	15185	TTATA	2	
69	P0000047	Potential SSR	PentaSSR	15347	15356	AATAA	2	
70	P0000049	Potential SSR	PentaSSR	15507	15516	AATTA	2	
71	P0000050	Potential SSR	PentaSSR	16581	16590	TAAAA	2	
72	H0000001	Potential SSR	HexaSSR	308	319	TATTTA	2	ND2
73	H0000002	Potential SSR	HexaSSR	3250	3261	TCAATT	2	COX2
74	H0000003	Potential SSR	HexaSSR	3742	3753	AAATTA	2	
75	H0000004	Potential SSR	HexaSSR	3937	3948	AATTTT	2	ATP6
76	H0000005	Potential SSR	HexaSSR	4151	4162	GTTTCA	2	ATP6
77	H0000006	Potential SSR	HexaSSR	4412	4423	TTAATT	2	ATP6
78	H0000007	Potential SSR	HexaSSR	4665	4676	TTAAGA	2	COX3
79	H0000009	Potential SSR	HexaSSR	7253	7264	ATAAAT	2	ND5
80	H0000010	Potential SSR	HexaSSR	8279	8290	AAGTTA	2	ND4
81	H0000011	Potential SSR	HexaSSR	8559	8570	AAATAT	2	ND4
82	H0000012	Potential SSR	HexaSSR	8643	8654	AAAATA	2	ND4
83	H0000013	Potential SSR	HexaSSR	8690	8701	GTAAAG	2	ND4
84	H0000014	Potential SSR	HexaSSR	9308	9319	AATAAA	2	ND4L
85	H0000015	Potential SSR	HexaSSR	9356	9367	AACTAA	2	ND4L
86	H0000016	Potential SSR	HexaSSR	9643	9654	TTATAT	2	ND6
87	H0000017	Potential SSR	HexaSSR	9759	9770	ATTTTC	2	ND6
88	H0000018	Potential SSR	HexaSSR	9882	9893	TTAATT	2	ND6
89	H0000020	Potential SSR	HexaSSR	10097	10108	AAAATT	2	
90	H0000021	Potential SSR	HexaSSR	10502	10513	TAAAAG	2	CYTB
91	H0000022	Potential SSR	HexaSSR	11303	11314	TAAATA	2	
92	H0000023	Potential SSR	HexaSSR	12259	12270	AAAATT	2	ND1
93	H0000024	Potential SSR	HexaSSR	13564	13575	ATAAAT	2	
94	H0000028	Potential SSR	HexaSSR	16555	16566	AAAATT	2	
95	He0000001	Extended SSR	HeptaSSR	11103	11116	AAATTTA	2	CYTB
96	He0000002	Extended SSR	HeptaSSR	12201	12214	AAAACCC	2	ND1
97	He0000003	Extended SSR	HeptaSSR	12501	12514	AAAATTA	2	
98	He0000004	Extended SSR	HeptaSSR	14470	14483	AATTTTT	2	
99	He0000005	Extended SSR	HeptaSSR	16468	16481	AAAATTT	2	
100	O0000002	Extended SSR	OctaSSR	11397	11412	TAAAAAAA	2	ND1

101	O0000003	Extended SSR	OctaSSR	16532	16547	AAAAAATT	2	
102	N0000001	Extended SSR	NonaSSR	7702	7719	ATAAAAAAA	2	ND5
103	De0000001	Extended SSR	DecaSSR	1001	1020	TTTTATCTAT	2	ND2
